# Fatty acids profile in Awassi sheep milk affected by some genes’ single and combined effects

**DOI:** 10.5455/javar.2025.l891

**Published:** 2025-03-25

**Authors:** Khaleel I. Jawasreh, Dana Khrais, Muhammad Alu’datt, Nizar J. Haddad, Sami Awabdeh, Mohammad Isam AlMadani, Mohamad Brake, Mohamad Ahmad Al-Araishi, Monther Sadder, Ahmad Al-Amareen

**Affiliations:** 1 Faculty of Agriculture, Jordan University of Science and Technology (JUST), Irbid, Jordan; 2 National Agriculture Research Center (NARC), Baqa`a, Jordan; 3 Innovation and Business Development, Fresh Del Monte, Amman, Jordan; 4 Qatar University, Qatar University Press, Qatar University, Doha, Qatar; 5 Science Faculty, Jerash University, Jerash, Jordan; 6 Faculty of Agriculture, The University of Jordan, Amman, Jordan

**Keywords:** Awassi ewes, fatty acids, gene interaction

## Abstract

**Objective:**

This study intended to figure out the effects of fixed effects and genes such as beta-lactoglobulin (β-LG), prolactin (PRL), annexin A9 (ANXA9), and acetyl-CoA acyltransferase 2 (ACAA2) on the structure of fatty milk acids in Awassi ewes, as well as any potential genotype-genotype interactions.

**Materials and Methods:**

Fatty acid (FA) profile and other milk components were examined from 116 Awassi ewes in total. Polymerase chain reaction (PCR) was used to extract and genotype their DNA, and either sequencing or restriction fragment length polymorphism (RFLP) analysis came next.

**Results:**

Saturated, medium-chain, and long-chain FA proportions were not significantly impacted by the genotypes of β-LG, PRL, ANXA9, and ACAA2. Conversely, ewes with the β-LG AA genotype displayed higher levels of monounsaturated fatty acids (MUFAs), while the β-LG AB genotype was linked to the highest levels of polyunsaturated fatty acids (PUFAs). It has been demonstrated that PUFA levels are impacted by single nucleotide polymorphisms (SNPs) of ANXA9, while VLCFA and PUFA levels are changed by SNPs of ACAA2. The PRL AA genotype showed the greatest influence on VLCFA. Furthermore, milk exhibited the highest levels of MUFA and PUFA due to the interaction between β-LG and PRL. The interaction PRL-ANXA9 exhibited the greatest levels of VLCFA. Additionally, Dam lambing weight affected the levels of MUFA, MCFA, and long-chain fatty acids.

**Conclusion:**

To increase the levels of MUFA and PUFA constituents, the selection program should effectively harness and integrate the synergistic benefits of β-LG, PRL, ANXA9, and ACAA2 as well as their interaction.

## Introduction

Nowadays, with functional food, sheep milk formulae can improve human health. Health-conscious people are choosing natural foods over drugs, which can have healthy side effects. Awassi sheep have become nomadic via natural and careful breeding over several hundred years. Jordan’s Awassi breed of sheep provides a lot of milk and is important in semi-arid regions in the East of the kingdom. Milking is easier with this quiet and easy-to-handle breed. The breed’s hardiness, grazing aptitude, grazing-based production suitability, and limited management are advantages. The chemical composition of Awassi sheep’s milk is 4.62% fat, 10.7% solid-non-fat, 4.66% lactose, and 5.4% protein [1].

The nutritional value of dairy products is impacted by the quality of the milk fat. These contain significant contents of monounsaturated fatty acids (MUFAs) and polyunsaturated fatty acids (PUFAs), particularly n-3 fatty acids (FAs), conjugated linoleic acid (CLA) n-6, and fat-soluble vitamins. Moreover, milk fat adds flavor and fragrance and aids in the processing of raw materials. In terms of fat chemical structure, there are over 400 chain structures of the FA [2].

Sheep milk fat contains the highest values of C4, C16, and C18 fatty acids. Ceyhan et al. [3] stated that the contents of solid non-fat (SFA), MUFA, and PUFA in Awassi sheep are 74.031%, 21.865%, and 3.078%, respectively. The presence of free butyric acid in milk was found to correlate with the inhibition of human cancer cell lines. Caproic, caprylic, and capric acids have the potential to decrease body fat and weight. About 80% of US disease-related deaths are caused by cardiovascular disease, cancer, obesity, and diabetes. Disease onset and progression are influenced by lipid contents. Dietary lipid composition and quantity are important factors in health prospects [4].

The fatty acid content in cow’s milk is particularly susceptible to change. Many factors affect the fat composition of milk, including breed, lactation stage, nutrition, parity, seasonal variation, and age. Furthermore, previous investigations have shown that genetic diversity affects the prevalence of specific types of fatty acids, particularly those produced from de novo mammary fatty acid production. Distinct breeds have distinct milk fatty acid profiles. Various dairy sheep breeds proposed putative genes influencing the composition of fatty acids and milk fat, such as β-LG, PRL, Annexin 9 (ANXA9), and ACAA2 [4].

The β-LG gene produces beta-lactoglobulin, which is the main whey protein of cow milk. Mammary gland secretory cells produce β-LG, which has been widely researched for its interaction with hydrophobic ligands, including vitamins and fatty acids. A previous study conducted by Kontopidis et al. [6] found that the β-LG is similar to retinol and fatty acids *in vitro*. Thus, β-LG may be a retinol-binding protein in a newborn’s intestinal tracts. It may also help pregastric lipase transport and metabolize dietary lipids by sequestering free fatty acids. Tsartsianidou et al. [7] discovered that chromosomes 3 and 11 in sheep, goats, and cows have β-LG mapping.

The prolactin gene (PRL gene) encodes a hormone that regulates lactation, osmoregulation, and reproductive activities. Prolactin, a lactogenic hormone, may be depleted during lactation and can restrict or prohibit milk production in many animals. The sheep prolactin gene has five exons and four introns on chromosome 20. The ovine prolactin gene has a polymorphism with two variations (A and B). PRL produces lipids, proteins, and other essential nutrients found in milk [8].

The Annexin gene produces ANXA9, which binds to phospholipids and calcium ions. It also helps the mammary gland transfer fatty acids across the cytoplasmic membrane [9]. Secretory tissue and mammary glands express the ANXA9 gene. Pecka-Kielb et al. [10] indicated a relationship between sheep’s milk composition and three particular single nucleotide polymorphisms (SNPs) in the ANXA9 gene on chromosome 1.

A genomic region on chromosome 23 where sheep have genomic loci for milk, protein, and fat yields contains the gene ACAA2, which catalyzes the final stage of fatty acid beta-oxidation. ACAA2’s 3’ untranslated region has an SNP that was associated with dairy Chios sheep milk, protein, and fat yield. The SNP displayed differential allelic expression [7].

The overall objective of this study is to examine the molecular information, sheep milk characteristics, and gene relationships in fatty acid groups in Jordanian commercial Awassi flocks. More specifically, the study aims to a) identify possible mutations in the β-LG, PRL, ANXA9, and ACAA2 genes in Awassi sheep; b) determine the frequency of these mutations in the flocks of Awassi sheep; and c) for the first time, we will investigate how these genotypes and their interactions affect the fatty acid composition.

## Materials and Methods

### Ethical approval

The Animal Care and Use Committee at Jordan University and Science Technology; approval Number 36/12/4/16 approved the procedures used in this experiment.

### Animal and sample collection

Milk was collected from Awassi ewes during their pre-weaning phase by trained technicians using a manual milking procedure. To ascertain the quantity of milk obtained during the nursing period, ewes were subjected to a daily milking procedure. After lambs weaning, the dams were milked twice daily for 60 days; once in the morning and once in the evening. The separation of lambs from their dam occurred 12 h before the beginning of morning milking. Ewes were deemed to have entered the dried-off lactation stage when their milk production declined to below 100 ml. The overall duration of lactation, covering both the suckling and milking phases, was computed. A method for determining the yield of each ewe’s test-day milk (TDM) was identified as the technique outlined by Jawasreh et al. [5].

### Data collection

A total of 1,133 milk samples were collected to gain an overview of the milk and its constituents. In the meantime, 120 Awassi ewes provided milk and blood samples, respectively, which were used in the fatty acid analysis. Following post-lambing, specifically at the mid-lactation stage (days ± standard error; 44 ± 3.14 days), the milk samples under analysis were obtained. They were then cooled and stored at −20°C until additional laboratory analysis.

The milk scan machine (Minor Type 78100, FOSS Electric, Hillerod, Denmark) was utilized to analyze the fundamental constituents of milk, including fat, protein, solid non-fat (SNF), lactose, and other milk components of 1,133 milk samples collected between 2019 and 2022.

### Fat extraction

Fat extraction and fatty acid analysis were performed at Military Quality Control Laboratories (Amman, Jordan) using the Gerber method for fat extraction for 118 samples that were described by Jawasreh et al. [5]. From a well-mixed aliquot of sheep’s milk, 10 ml was placed in a butyrometer (Gerber) tube with 10 ml of 90% sulfuric acid, (1:1) milk and acid ratio for 15 min, and then the isoamyl alcohol was added gradually to complete the volume of the tube. The mixture was centrifuged at 350 rpm for 10 to 15 min at 65°C in centrifuge AXIOM mini/F15. The fat layer was suctioned from the Gerber tube.

### Fatty acids (FA) analysis

The FA composition was determined through the process of transesterification, which involved the conversion of fatty acids into methyl esters (fatty acid methyl esters). The preparation of methyl esters involved the utilization of a solution of fat in heptane (0.1 gm in 2 ml), which was subjected to intense agitation with 0.2 ml of 2 mol KOH in methanol within a test tube that was equipped with a screw cap. Following centrifugation, the supernatant layer was subjected to immediate gas chromatography (GC) analysis after filtration through polytetrafluoroethylene membrane filters (Millex-FG, 13 mm). The analysis of fatty acid methyl esters was conducted through the employment of a gas chromatograph (SHIMADZU GC for mass spectrometer GC-2010 plus, GCMS-TQ8040 NX GC-MS/MS, Kyoto, Japan) that was equipped with a fused silica capillary column db-wax (SP-2380, 100 m × 0.25 mm; Agilent, USA). Helium was utilized as a carrier gas, injector, and flame ionization detector (FID SHIMADZU, Kyoto, Japan). A set of 18 fatty acids was utilized in conjunction with purified individual fatty acids to establish standard retention times. Fatty acids were identified through the analysis of their retention times with those fatty acids present in standard samples. Then, we classified the 18 fatty acid found in the analysis as SFA, MUFA, PUFA, MCFA, long-chain fatty acids (LCFAs), and VLCFA.

### DNA extraction and amplification process

MasterPure DNA Purification Blood Kit (GeneAll Exgene, Korea) was used for DNA isolation, following the manufacturer’s recommendations. The animal genotyping process was conducted using restricted fragment length polymorphism PCR-RFLP that was adopted after the amplification process of the targeted genomic region; thereafter, the Sanger sequence analysis was adopted for identifying the nucleotides in each product. The Primer3 tool (http://bioinfo.ut.ee/primer3–0.4.0/) was used to design the primers, and Table 1 displays the locations of each SNP [11].

Using a 2× PCR Master Mix (Applied Biological Materials Inc. (ABM), Canada) with a certain amount of the genomic DNA (50 ng) and 5 pmol of each primer, the PCR was carried out in a final volume of 20 μl. An initial denaturation step of 3 min at 95°C was followed by 30 cycles of 15 sec denaturation at 95°C each, followed by 15 sec of annealing at a temperature suitable (Table 1) for each genomic region, 15 sec at 72°C (extension step), and then using the same temperature for 1 min at 72°C (final extension).

### Sequencing analysis

The sequencing method was applied using the primers included in PCR (Macrogen Incorporation in Seoul, South Korea) and then cleaned and sequenced using the PCR genotyping patterns of the ANXA9 and ACAA2 to detect SNPs. To confirm the results from the PCR-RFLP technique, the sequences of 116 samples were looked at both ways and compared with the reference sequence from NCBI (National Center for Biotechnology Information). BioEdit program version 5.0.6 was used to conduct sequence analysis and alignments [12].

**Table 1.** Primer sequences, their location, the annealing temperature used, and the product size.

Gene	Location	Primer sequence (5'–3')	Annealing Temperature (°C)	PCR Product (bp)
ANXA9 chromosome 1 ([15]	Exon 4 Intron 5	F: CATTCCTGTGTGTCCGGTAC R: TCATCTCAGACCTAACCACCA	50	675
ACAA2 chromosome 23	Intron 2 Exon 2 Intron 3	F: AAGCCTGCCAAGCAGTTCT R: TTCCCCACAACAAACACTGA	58	720
Beta-lactoglobulin (β-LG)	Exon 2	F:CTCTTTGGGTTCAGTGTGAGTCTTG R: CACCATTTCTGCAGCAGGATCTC	58	301
Prolactin (PRL)	Intron 2	F: ACCTCTCCTCGGAAATGTTCA R: GGGACACTGAAGGACCAGAA	56	1209

### RFLP analysis

The identification of variations in the β-LG and PRL genes was accomplished using the PCR-RFLP technique with a final volume of 10 μl of PCR product, 5 μl of buffer, 2 μl of 1× BSA, 2 μl of nuclease-free water, and 2 μl of restriction enzyme. The 301 bp amplified β-LG gene fragment underwent digestion using the RsaI restriction enzyme at a temperature of 37°C for 1 h. The restriction products were subjected to gel electrophoresis using an agarose gel (2%) containing ethidium bromide, followed by visualization using a gel documentation system that adopted UV. The PRL gene fragment, which was amplified and measured to be 1209 bp, underwent restriction using the HaeIII endonuclease enzyme at a temperature of 37°C for 1 h. The visualization of the RFLP profile was conducted using the same methodology as that employed for β-LG.

### Statistical analysis

The population genetics measures of the ANXA9, ACAA2, and PRL in addition to the β-LG loci were generated according to Falconer and Mackay’s [13] procedure. The chi-square statistical method was conducted to assess the degree of conformity of the studied population. The study employed the least-squares method within a mixed-model framework to examine the impact of parity, type of birth, ANXA9, ACAA2, β-LG, and PRL genotypes, as well as their potential interactions, on the observed phenotypes. The analysis was conducted using the online version of SAS On Demand for Academics software in June 2023.

The factors that did not significantly affect the studied traits or even affect the impact of the other factors were removed from the model after several statistical models had been investigated using all available factors (Parity, Age, Dam weight at lambing, type of birth) considering that the samples were taken in the middle stage of the lactation period, implying the elimination of the lactation phase., the analysis of the milk fatty acid groups was conducted using the final fit model as follows:

Yijklnmopqr = μ + PRL_i_+ β-LG_ j_ + (β-LG × PRL) _ij_ + A91_k_ + A92_l_ + A93_m_ + A95_n_ + A21_o_ + A23p + A24_q_ + (PRL × A24) _iq_+ (PRL × A95) _jn_ + (PRL × A93) _im_ + WLr + e_ijklmnopqr_

Where:

- Yijklnmopqr = the studied traits SFA, MUFA, PUFA, MCFA, LCFA and VLCFA;

- μ = overall mean of the milk composition or fatty acids groups;

- β-LG i = fixed impact of the ith genotype at β-LG genomic region (I = AA, AB and BB);

- PRLj = fixed impact of the jth genotype at PRL genomic region (j = AA and BB);

- A91k = fixed impact of the kth genotype at ANXA9 1 genomic region (k = GG, GC and CC);

- A92l = fixed impact of the lth genotype at ANXA9 2 genomic region (l = GG, GT and TT);

- A93m = fixed impact of the kth genotype at ANXA9 3 genomic region (m = GG, GC and CC);

- A95n = fixed impact of the nth ANXA9 5 locus (*n* = GG, GT and TT);

- A21o = fixed impact of the oth genotype at ACAA2 1 genomic region (o = GG, AG and AA);

- A23p = fixed impact of the pth genotype at ACAA2 3 genomic region (p = GG, GT and TT);

- A24q = fixed impact of the pth genotype at ACAA2 4 genomic region (p = CC and AA);

- (β-LG × PRL)ij = interaction effect of β-LG and PRL genotypes (ij = AAAA,

AABB, ABAA, ABBB, BBAA, and BBBB);

- (PRL × A24)iq = interaction between PRL genotypes and ACAA2 4 genotypes (ik = AACC, AAAA, BBCC and BBAA);

- (PRL × A95) jn = interaction between PRL genotypes and ANXA9 5 genotypes (jk = AAGG, AAGT, AATT, BBGG, BBTT, BBGT);

- (PRL × A93) im = interaction effect of PRL and ANXA9 3 genotypes (jk = AAGG, AAGC, AACC, BBGG, BBGC and BBCC);

- WLr = regression coefficient dam weight at lambing

- Eijklmnopqr = random residual with the assumption of *N* (0, *σ*2).

The model excluded the four-way interaction effect involving the five genes due to their lack of statistical significance. Statistical comparisons were deemed statistically significant if the probability level (*p*-value) was less than 0.05.

## Results

The descriptive statistics mean coefficient of variation and standard error for the contents of milk production traits represented by TDM yield, milk composition traits (fat, SNF, protein, density, and lactose), 6 groups of FA (SFA, MUFA, PUFA, MCFA, LCFA, and VLCFA), and atherogenic index studied are shown in Table 2.

The average fat and protein levels in Awassi sheep were found to be 5.593 ± 0.059 and 4.549 ± 0.013, respectively. The recorded values for SNF and lactose are 9.647% and 4.347%, respectively. The average values for the several classes of fatty acids were 65.4%, 31.73%, 2.77%, 11.6%, 88.1%, and 0.26% for SFA, MUFA, PUFA, MCFA, LCFA, and VLCFA.

**Table 2.** Descriptive statistics of TDM, milk composition traits, and fatty acids groups.

Variable	*N*	Mean	Coefficient of variation (%)	Std Error	Minimum	Maximum
TDM (kg)	1133	1.048	39.027	0.012	0.211	3.172
Fat (%)	1132	5.593	35.437	0.059	2.050	15.400
SNF (%)	1131	9.647	9.174	0.026	6.200	14.250
Density (g/ml)	1131	33.357	12.426	0.123	20.000	46.400
Protein (%)	1131	4.549	9.760	0.013	2.900	6.800
Lactose (%)	1131	4.347	9.708	0.013	2.800	6.420
SFA (%)	117	65.394	8.591	0.519	46.300	79.220
MUFA (%)	117	31.727	18.521	0.543	19.020	49.830
PUFA (%)	117	2.769	66.053	0.169	0.410	9.830
MCFA (%)	117	11.587	33.512	0.359	2.360	22.780
LCFA (%)	117	88.049	4.617	0.376	76.010	97.830
VLCFA (%)	117	0.255	40.784	0.010	0.120	1.200
AI	117	2.258	31.577	0.066	0.624	5.100

TDM: test-day milk, SNF: solid non-fat, SFA: saturated fatty acids, MUFA: monounsaturated fatty acids, PUFA: polyunsaturated fatty acids, MCFA: medium chain fatty acids, LCFA: long chain fatty acids, VLCFA: very long chain fatty acids, AI: atherogenic index.

**Figure 1.**
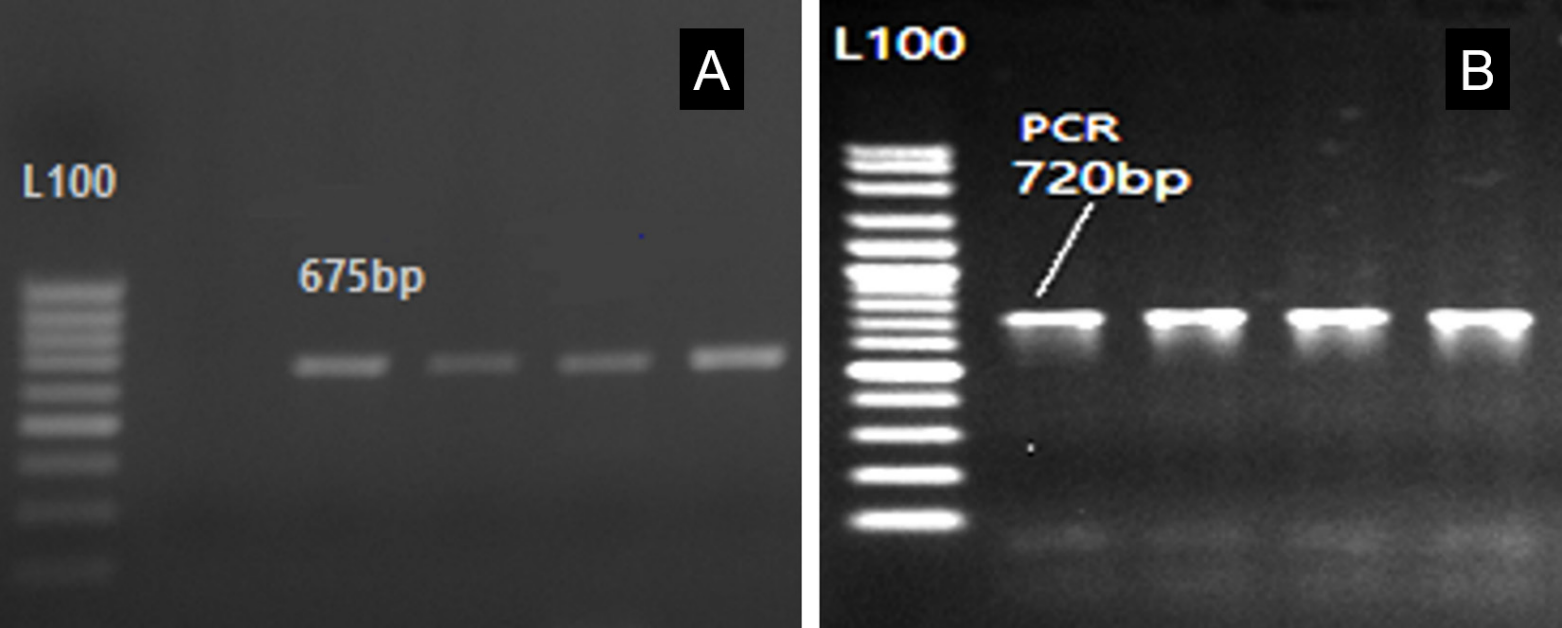
The PCR product band of the ANXA9 gene of 675 bp in size and 720 bp for the ACAA2 gene in size, L: 100 bp DNA Ladder.

**Figure 2.**
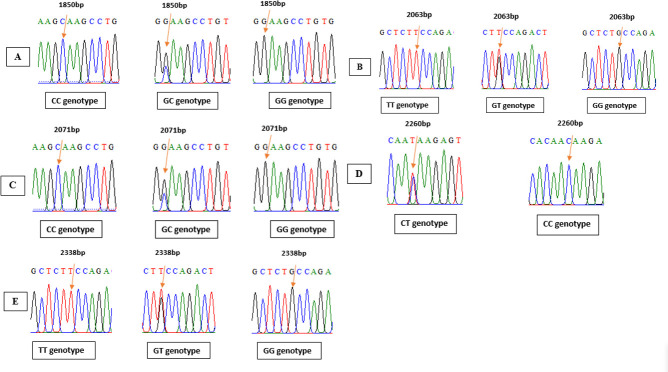
The location of the Awassi sheep’s ANXA9 gene variations, and the nitrogenous bases sequences of the ANXA9 gene.

As observed in Figure 1, the ANXA9 genetic region was effectively amplified, yielding a 675 bp product. The PCR results for the ANXA9 and ACAA2 genes were sequenced using certain primers, and the Bio Edit software was used to align and analyze the sequences [12]. In addition, five mutations of ANXA9 were detected: the first one, C > G, located at 1,850 bp (Fig. 2A)*; *the second one, T > G, was identified at the 2063 bp position (Fig. 2B); and the C > G mutation was found at the 2,071 bp (Fig. 2C). Another mutation, C > T was detected at the 2,260bp location (Fig. 2D). In conclusion, as shown in Figure 2E, a G > T mutation was observed at the 2,338 bp position relative to gene sequence size.

**Figure 3.**
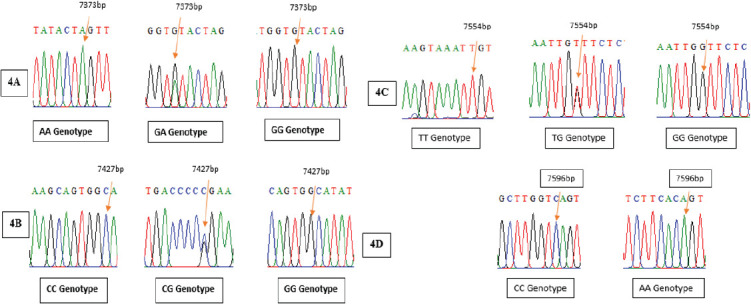
The location of the Awassi sheep’s ACAA2 gene variations, as well as the nitrogenous bases sequences of the ACAA2 gene.

**Figure 4.**
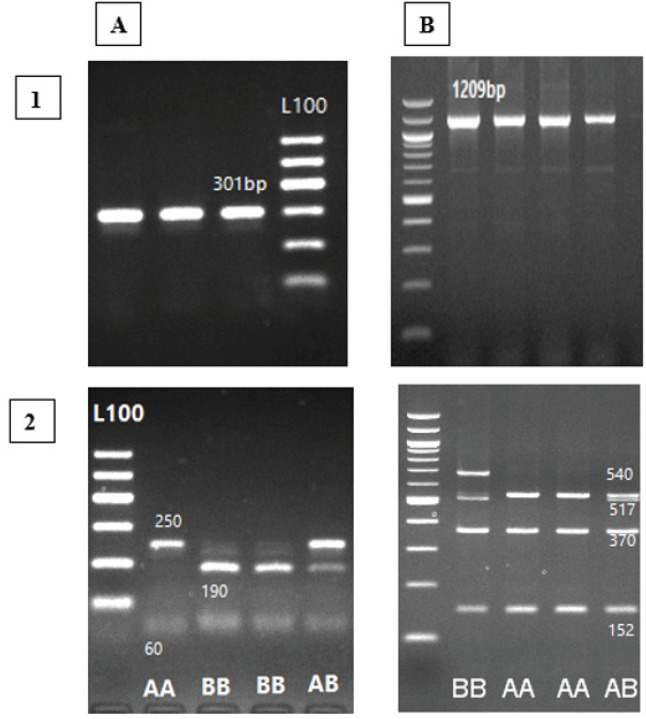
PCR-RFLP results for β-LG (A) and prolactin (B) genes using RsaI and HaeIII restriction enzymes, respectively on 2% agarose gel. Panel A, A1: PCR product, A2: Lanes 1, 2 and 3 are: AB (250, 175, 190, and 60 bp), AA (250 and 60 bp), and BB (190 and 60 bp) genotypes, and L100: Ladder 100 bp. Panel B, B1:PCR product, B2: Lanes 1, 2 and 3 are: BB (517, 370 and 152 bp), AA (540, 370 and 152 bp), and AB (540, 517, 370 and 152 bp) genotypes, and L100: Ladder 100 bp.

The amplification of the 720 bp fragment of the ACAA2 gene was amplified successfully, as shown in Figure 1. Four mutations in the ACCA2 gene were found by the investigation. At position 7373bp, mutation G < A (rs400473337) specifically displayed three genotypes (GG, GA, and AA) (Fig. 3A). Figure 4B illustrates how three genotypes (CC, CG, and GG) were present at location 7427 bp for another mutation, C < G (rs425846296). Furthermore, three genotypes (TT, TG, and GG) were revealed by the mutation G < T (rs410726616) at position 7554 bp (Fig. 3C). Finally, Figure 3D displays two genotypes (CC and AA) at location 7596 bp associated with a mutation that had not yet been discovered. Moreover, the positioning of all mutations is dependent upon the size of the gene sequence.

The amplification of the β-LG gene fragment was achieved through the utilization of the PCR technique, yielding a single product of 301 bp. The utilization of the RsaI restriction enzyme in the process of digestion resulted in the identification of three distinct genotypes, which have been categorized as AB (250, 190, 60 bp), AA (250, 60 bp), while BB was of two bands of 190 and 60 bp (Fig. 4-A2). The amplification of the 1209 bp fragment for the PRL gene was achieved successfully. Figure 4 -B2 shows how the HaeIII endonuclease restriction enzyme breaks down the PCR-amplified PRL gene. This analysis presented the presence of two distinct alleles, namely A and B. These alleles were found to exist in three different genotypes, each of which was characterized by varying fragment sizes. The genotypes identified were AA (with fragment sizes of 540, 370, and 152 bp), AB (with fragment sizes of 540, 517, 370, and 152 bp), and BB (with fragment sizes of 517, 370, and 152 bp).

Three genotypes (CC, GC, and GG) of the first mutation of the ANXA9 gene were of observed frequencies of 0.59, 0.37, and 0.03, respectively. The allele frequencies were 0.78 and 0.22, respectively, for C and G alleles. The second mutation was found to have three genotypes (TT, GT, and GG) with observed genotype frequencies of 0.57, 0.39, and 0.04, respectively. For the T and G alleles, the frequencies of different alleles were 0.76 and 0.24, respectively. Furthermore, the frequencies of the three genotypes of the third mutation (CC, GC, and GG) are 0.28, 0.53, and 0.18, respectively, while allele frequencies of C and G alleles were 0.55 and 0.45, respectively. The frequencies of the fourth mutation’s two genotypes (CC and CT) were found to be 0.97 and 0.03, respectively. The C and T alleles had observed frequencies of 0.99 and 0.01, respectively. The final mutation had three genotypes (GG, GT, and TT), each with a frequency of 0.89, 0.06, and 0.05. The frequencies of the G and T alleles were 0.92 and 0.08, respectively, as seen in Table 3.

The first mutation of the ACAA2 gene exhibits three distinct genotypes (GG, GA, and AA), which had been observed with frequencies of 0.65, 0.28, and 0.07, respectively. The observed frequencies of the G and A alleles were 0.79 and 0.21, respectively. Also, the frequencies of the three genotypes of the second mutation (CC, GC, and GG) were 0.65, 0.28, and 0.07, respectively. The frequencies of alleles C and G were 0.79 and 0.21, respectively. The third mutation demonstrates three genotypes (GG, GT, and TT), which have been observed at frequencies of 0.71, 0.24, and 0.05, respectively. The frequencies of the G and T alleles were observed to be 0.84 and 0.16, respectively. Only two genotypes (CC and AA) were found for the fourth mutation, with observed frequencies of 0.92 and 0.08, respectively. For alleles C and A, the corresponding frequencies were 0.92 and 0.08. While the genotypic frequencies of BB, AB, and AA were 0.72, 0.01, and 0.27, respectively, the allele frequencies of the PRL genotypes were 0.72 and 0.28 for the B and A alleles. Three genotypes (AA, AB, and BB) were also identified for the β-LG gene, with observed frequencies of 0.26, 0.45, and 0.29, respectively. The A and B alleles of the β-LG gene had respective allelic frequencies of 0.48 and 0.52.

**Table 3.** Variant detected within ANXA9, ACAA2, PRL, and β-LG, their gene and genotype frequencies, and Hardy Weinberg Equilibrium (HWE) in the studied Awassi.

Gene	Genotype	Observed number	Expected number	Genotype frequency	Allele	Allele frequency	Value of *x*^2^ test
ANXA9 First mutation	GG	4	5.61	0.03	G	0.22	0.755
	GC	43	39.81	0.37	C	0.78	
	CC	69	70.57	0.59			
ANXA9 Second mutation	GG	5	6.68	0.04	G	0.24	0.609
	GT	45	42.3	0.39	T	0.76	
	TT	66	67	0.57			
ANXA9 Third mutation	GG	21	23.49	0.18	G	0.45	0.75
	GC	62	57.42	0.53	C	0.55	
	CC	33	35.09	0.28			
ANXA9 Fourth mutation	CT	3	2.29	0.03	C	0.99	0.235
	CC	113	113.69	0.97	T	0.01	
ANXA9 Fifth mutation	GG	103	98.18	0.89	G	0.92	43.55
	GT	7	17.07	0.06	T	0.08	
	TT	6	0.74	0.05			
ACAA2 First mutation	GG	75	72.39	0.65	G	0.79	2.5
	GA	33	38.48	0.28	A	0.21	
	AA	8	5.116	0.07			
ACAA2 Second mutation	CC	75	72.39	0.65	C	0.79	2.5
	CG	33	38.48	0.28	G	0.21	
	GG	8	5.116	0.07			
ACAA2 Third mutation	GG	83	81.84	0.71	G	0.84	1.73
	GT	28	31.18	0.24	T	0.16	
	TT	5	2.97	0.05			
ACAA2 Fourth mutation	CC	107	98.18	0.92	C	0.92	110.1
	AC	0	17.1	-	A	0.08	
	AA	9	0.74	0.08			
PRL	AA	32	9.17	0.27	A	0.28	110.97
	AB	1	47.17	0.01	B	0.72	
	BB	84	60.65	0.72			
β-LG	AA	30	26.96	0.26	A	0.48	1.0198
	AB	53	58.41	0.45	B	0.52	
	BB	34	31.64	0.29			

Table 4 presents the statistical findings indicating that the studied genes, PRL and ACAA2-4, and their interaction have a statistically significant (*p* < 0.05) impact on VLCFA. The interaction between the β-LG gene and the combined gene genotypes of β-LG and PRL exhibited a statistically significant impact on the levels of MUFA and PUFA. In addition, it was shown that the contents of PUFA were influenced by the presence of ANXA9-3, ANXA9-5, and ACAA2-1, as well as the combined effect of PRL and ANXA9-3 (*p* < 0.05). Nevertheless, no significant impact of the single genes and their interactions on MCFA and LCFA (*p* > 0.05). Conversely, the decline in the weight of the dam at lambing exhibited a noteworthy impact on the levels of MUFA, MCFA, and LCFA (*p* < 0.05).

**Table 4.** *p-values* for the effect of certain factors including genomic regions with interactions on fatty acid groups.

Factors	MUFA gm/100 gm Fat	PUFA gm/100 gm Fat	MCFA gm/100 gm Fat	LCFA gm/100 gm Fat	VLCFA gm/100 gm Fat
PRL	0.825	0.126	0.890	0.724	0.013
β-LG	0.013	0.004	0.193	0.407	0.764
PRL* β-LG	0.004	< 0.0001	0.347	0.512	0.809
A91	0.646	0.719	0.822	0.799	0.853
A92	0.658	0.753	0.622	0.648	0.910
A93	0.361	0.021	0.584	0.101	0.909
A9	0.486	0.001	0.440	0.250	0.870
A21	0.480	0.019	0.555	0.624	0.853
A23	0.600	0.144	0.860	0.689	0.716
A24	0.548	0.559	0.555	0.477	<0.0001
PRL*A24	0.603	0.318	0.423	0.455	<0.0001
PRL*A95	0.904	0.281	0.587	0.515	0.891
PRL*A93	0.302	0.036	0.946	0.217	0.944
WL	<0.0001	0.153	0.008	0.023	0.552

MUFA = monounsaturated fatty acids; PUFA = polyunsaturated fatty acids; MCFA = medium chain fatty acids; LCFA = long chain fatty acids; VLCFA = very long chain fatty acids; PRL = prolactin; β-LG = beta-lactoglobulin; A91 = Annexin A9 first mutation; A92 = Annexin A9 second mutation; A93 = Annexin A9 third mutation; A95 = Annexin A9 fifth mutation; A21 = Acetyl-CoA Acyltransferase 2 first mutation; A23 = Acetyl-CoA Acyltransferase 2 third mutation; A24 = Acetyl-CoA Acyltransferase 2 fourth mutation; WL = weight of lambing. The * sign means the interaction between the genes.

Table 5 shows the effect of β-LG and its interaction with PRL genotypes on MUFA. The AA genotype of the β-LG gene was associated with the highest MUFA levels (36.36%), while AB and BB genotypes were of the lowest MUFA (31.35% and 31.31%, respectively) (*p *< 0.05). The interaction of AA × AA genotype (PRL* β-LG) showed the highest MUFA levels (39.62%), while the interaction of AA × AB and AA × BB genotypes of PRL* β-LG showed the lowest levels (29.77% and 28.53%, respectively) (*p* < 0.05). The AB genotype of β-LG recorded the highest PUFA% (3.03 ± 0.61); also, the AB genotype individual showed a significant difference from the individuals carrying the AA genotype (1.29 ± 0.76), while there was no significant difference with the BB genotype (2.92 ± 0.67). The AA × AA genotype interaction of PRL* β-LG showed the lowest PUFA levels (0.97 ± 1.19) and significantly differed from the other genotypes. The ANXA9-3 CC genotype was linked with the highest PUFA levels, and it had a significant difference with the GG genotype and a non-significant difference with the GC genotype (1.33 ± 0.78 and 2.87 ± 0.66, respectively). Moreover, the GG genotype of ANXA9-5 and AA genotype of ACAA2-1 were connected with the highest levels of PUFA (4.50 ± 0.85 and 3.57 ± 0.72, respectively). The highest PUFA percentage was found in the BB × GC genotypic interaction between PRL & ANXA9-3 (4.05 ± 0.69), and that had a significant difference with other genotypes of that interaction.

The effect of PRL and ACAA2-4 genotypes on VLCFA% is shown in Table 6. The AA genotype of PRL and ACAA2-4 showed the highest levels of VLCFA (0.43 ± 0.05 and 0.44 ± 0.05, respectively), compared to the other two genotypes of the two genes (*p* < 0.05). The regression of the weight of the dam at lambing affected significantly MUFA, MCFA, and LCFA 0.31 ± 0.07, −0.13 ± 0.05, and 0.12 ± 0.05, respectively (Table 7).

## Discussion

This investigation confirmed the association between β-LG, PRL, ANXA9, and ACAA2 gene variants and their interaction and fatty acid groups in Awassi sheep’s milk. The selection of these genes was based on their direct role in the mammary gland development and growth, milk synthesis, fat production, and fatty acids. The genes are additionally situated within the genomic region of QTL that exert an influence on both the amount and quality of milk [1]. The investigation indicated that allelic frequencies and genotypes of these genes exhibit variations in Awassi sheep.

The ANXA9 gene exhibited greater frequency in the locus that hosts the C allele (0.78) versus the G allele (0.22) in its first mutation (C > G). Similarly, the second mutation of the gene (T < G) displayed a higher frequency of the T allele (0.76) than the G allele (0.24), while the C allele had a higher frequency (0.55) than the G allele (0.45) in the mutation detected in C > G. On the other hand, the highest frequency was (0.99) for the C allele in the C > T mutation located on 2260 bp. Finally, the last mutation (G > T) demonstrated a frequency that was higher for the G allele (0.92) in contrast to the T allele (0.08) in Awassi sheep (Table 3). The study conducted by Almaamory and Al-Anbari [14] revealed comparable findings about the occurrence of a second mutation (T < G) in Awassi sheep. The allele frequencies observed were 0.72 for allele T and 0.28 for allele G. According to Pecka-Kielb et al. [15], the study indicated the presence of three mutations identified by different restriction enzymes, namely NlaIII, HinfI, and Tru1. The first mutation (G < A) detected by NlaIII exhibited a higher frequency of the G allele (0.66) compared to the A allele (0.34). The second mutation (G < C) identified by the HinfI enzyme demonstrated a higher frequency of the G allele (0.54) and a lower frequency of the C allele (0.46). In Zošľachtená valaška sheep, the third mutation (C < A) that was detected by the Tru1 enzyme exhibited a higher frequency of the C allele (0.57) in comparison to the A allele (0.43). In the first mutation of ACAA2, the G allele frequency (0.79) was more than the A allele (0.21), while the C allele (0.79) was more than the G allele (0.21) in the second mutation. On the other hand, the G allele (0.84) was higher than the T allele (0.16) in the third mutation. In the fourth mutation, the C allele (0.92) was higher than the A allele (0.08); also, this mutation had been undiscovered before (Table 3). The allelic frequencies at the ACAA2 SNP locus in exon 10 for the C and T alleles, respectively, were found to be 0.47 and 0.53 according to Symeou et al.’s [16] study results in Chios sheep. This comes in line with the findings of [17], who found that the T and C alleles were 0.56 and 0.44, and [7], who observed that the C allele was 0.46 and the T allele was 0.54.

**Table 5.** Effect of Beta-lactoglobulin (β-LG) genotypes, significant interaction between PRL & β-LG, ANXA9 3, ANXA9 5, ACAA2 1, and significant interaction between PRL & ANXA9 3 effects on MUFA and PUFA polyunsaturated fatty acids in Awassi sheep.

Gene	Genotype	N	MUFA gm/100 gm fat least square means (± SE)	PUFA gm/100 gm fat least square means (± SE)
β-LG	AA	30	36.36 ± 2.54^a^	1.29 ± 0.76^b^
	AB	52	31.35 ± 2.03^b^	3.03 ± 0.61^a^
	BB	32	31.31 ± 2.23^b^	2.92 ± 0.67^a^
PRL* β-LG	AAAA	6	39.62 ± 3.97^a^	0.97 ± 1.19^b^
	AAAB	14	29.77 ± 2.94^b^	2.80 ± 0.88^a^
	AABB	10	28.53 ± 3.26^b^	3.10 ± 0.98^a^
	BBAA	24	33.10 ± 2.69^a^	3.56 ± 0.81^a^
	BBAB	38	32.94 ± 2.25^a^	3.27 ± 0.67^a^
	BBBB	22	34.09 ± 2.48^a^	2.74 ± 0.74^a^
ANXA9-3	CC	33	-	3.05 ± 0.63^a^
	GC	61	-	2.87 ± 0.66^a^
	GG	20	-	1.33 ± 0.78^b^
ANXA9-5	TT	6	-	2.18 ± 0.55^a^
	GT	7	-	0.56 ± 0.95^c^
	GG	101	-	4.50 ± 0.85^b^
ACAA2-1	GG	73	-	1.64 ± 0.83^a^
	AG	33	-	2.03 ± 0.76^b^
	AA	8	-	3.57 ± 0.72^b^
PRL*ANXA9-3	AACC	10	-	2.99 ± 0.93^ba^
	AAGC	15	-	1.68 ± 1.00^bc^
	AAGG	5	-	0.26 ± 1.17^c^
	BBCC	23	-	3.11 ± 0.75^ba^
	BBGC	46	-	4.05 ± 0.69^a^
	BBGG	15	-	2.40 ± 0.84^bc^

Within the same column different letters indicate significant differences *p* < 0.05. The * sign means the interaction between the genes.

The A allele frequency of the PRL gene (0.28) was higher than the PRL-B allele (0.72) in Awassi sheep, similar to Ozmen and Kul’s [18] findings in Awassi sheep that were 0.23 and 0.77 for A and B alleles, respectively. Similarly, the Akkaraman breed had frequencies of 0.15 and 0.85 for the A and B alleles, respectively. An allele frequency was 0.64 in Serra da Estrela, 0.57 in White Merino, 0.72 in Black Merino, and 0.64 in Spanish Merino sheep [19]. In Awassi sheep, the β-LG gene exhibited a prevalence of 0.52 to the B allele while the allele A was 0.48. Comparable findings were observed in the Rusty Tsigai breed, Chios sheep breed [18], and Awassi breed [4]. Falconer and Mackay [13] recognized mutations, migration, genetic drift, and selection as major factors influencing gene and genotype frequency.

**Table 6.** Effect of prolactin (PRL) genotypes and Acetyl-CoA Acyltransferase 2 fourth mutation (ACAA2 4) effects on VLCFA very long chain fatty acids in Awassi sheep.

Gene	Genotype	*N*	VLCFA gm/100 gm fat least square means (± SE)
PRL	AA	30	0.43 ± 0.05^a^
	BB	84	0.27 ± 0.04^b^
ACAA2-4	AA	9	0.44 ± 0.05^a^
	CC	105	0.26 ± 0.03^b^

PRL = prolactin; ACAA2-4 = Acetyl-CoA Acyltransferase 2 fourth mutation Within the same column different letters indicate significant differences *p* < 0.05.

**Table 7.** The effect of the weight of Dam at lambing (WL) on MUFA, MCFA, and LCFA in Awassi sheep’s milk.

Source	*N*	Regression on WL gm/Kg
MUFA gm/100 gm fat least square means (± SE)	115	0.31 ± 0.07
MCFA gm/100 gm fat least square means (± SE)	115	−0.13 ± 0.05
LCFA gm/100 gm fat least square means (± SE)	115	0.12 ± 0.05

The mean fat content was 5.593 ± 0.059, and the average protein content was 4.549 ± 0.013. In comparison with previous results, Zajác et al. [20] reported higher fat and protein percentages in the milk of Tsigai sheep’ (7.77% ± 1.606% and 5.94% ± 0.690%, respectively), improved Valachian sheep (7.48% ± 1.446% and 5.82% ± 0.620%, respectively), and Lacaune sheep (6.97% ± 1.514% and 5.62% ± 0.692%, respectively).

In a study conducted on Awassi sheep, the average lactose and SNF contents were found to be 4.35 and 9.65, respectively. In comparison, Wendorff and Haenlein [21] reported average values of lactose and SNF to be 4.06 and 11.18, respectively, in Chios sheep; 4.60 and 11.15, respectively, in Sarda sheep; 4.25 and 11, respectively, in Churra sheep; and 4.26 and 11.63, respectively, in Karaman sheep.

The following were the Awassi sheep’s mean values for various fatty acid categories: SFA made up 65.4% of the total, followed by MUFA (31.73%), PUFA (2.77%), MCFA (11.6%), LCFA (88.1%), and VLCFA (0.26%). According to Ceyhan et al. [3], the percentages of SFA, MUFA, and PUFA in Awassi sheep were found to be 74.031%, 24.82%, and 3.078%, respectively. Churra sheep exhibited an SFA percentage of 71.53, MUFA was 22.1%, PUFA was 6.54%, MCFA was 17.77%, and LCFA was 36.32%.

According to the study conducted by Ptacek et al. [22], a comprehensive examination of milk fat reveals a clear and statistically significant rise in lambing weight because of the augmentation of MUFA or PUFA. In contrast, a notable negative linear regression was observed with SFA. As stated by Abousoliman et al. [23], it can be inferred that fatty acids that are widely recognized for their positive health effects have a favorable influence on the growth abilities of lambs. Conversely, fatty acids are classified as “health risk” within the SFA group, specifically lauric acid (C12:0), myristic acid (C14:0), and palmitic acid (C16:0), which exhibited a negative association with lambs’ growth abilities. Here we can explain the correlation discussed by Falconer and Mackay [13], who indicated the crossing over and pleiotropic effects, as well as environmental effects that may link those traits with each other.

The final step of the fatty acid β-oxidation is largely impacted by the ACAA2 gene. Abousoliman et al. [24] revealed that this gene is in a part of the genome where QTLs are linked to sheep’s milk, fat, and protein yields. The amount of PUFA in Awassi sheep was most affected by the AA genotype of the ACAA2 gene at the 7373bp G < A (SNP) site. For the same sheep breed, the percentage of VLCFA was most affected by the AA genotype of ACAA2 at 7596 bp (C < A SNP). In their study, Symeou et al. [16] found that the presence of the ACAA2 g.2982 T > C (SNP) had negative dominance effects on the fatty acid (FA) components (C9:0, C11:0, C12:1 cis-9, and C13:0). Also, the FA index *ω*6/*ω*3 had a negative effect shown with ACAA2 variants. For the FA index, this SNP was responsible for 0.76% of the total trait difference between Chios sheep individuals. To the best of our understanding, there is limited research available on the association between ACAA2 and fatty acids in sheep’s milk. In our study, PUFA percentages were affected by ANXA9 (2071 bp C/G and 2338 bp G/T SNPs). The CC genotype of ANXA9 C/G and the GG genotypes had the most significant impact on PUFA%. The impact of ANXA9 variants on the structure of sheep’s milk, including the fatty acid profile, has only been briefly studied in the literature that is currently available. Bovine milk fat production and ovine milk fat contents are significantly impacted by the ANXA9 gene polymorphism. An SNP in the ANXA9 gene has been shown to affect the distribution patterns of numerous fatty acids, including C15:0, C18:0, C18:1 trans 9, C20:0, C20:4n6, and CLA, according to Pecka-Kielb et al. [9].

According to Pecka-Kielb et al.’s [15] findings in Zošľachtená valaška sheep, the ANXA9/HinfI polymorphism showed a substantial increase in pentadecanoic acid (C15:0) and eicosanoic acid (C20:0) contents in milk from homozygous CC genotype compared to heterozygous individuals (*p *< 0.05). The milk produced from sheep of the CG genotype had a significantly lower concentration of octadecanoic acid (C18:0) (*p* < 0.05) compared to the milk from animals with the homozygous GG genotype. ANXA9 polymorphisms did not significantly affect milk residual saturated fatty acids. The ANXA9/NlaIII polymorphism showed a substantial decrease (*p* < 0.01) in the proportion of (all-Z)-5, 8, 11, 14-eicosatetraenoic acid (20:4n6) in sheep milk among homozygous AA genotype individuals compared to heterozygous and homozygous GG genotypes. Homozygous sheep with the ANXA9/HinfI polymorphism had higher levels of trans-octadecenoic acid (C18:1n9t) in their milk compared to those with the CC genotype (*p *< 0.01) and heterozygous (*p *< 0.05). This study found a significant difference (*p* < 0.05) in the proportion of CLA detected in homozygous GG individuals compared to heterozygous individuals. The ANXA9/Tru1I polymorphism affected sheep milk C18:1n9t acid. Homozygous AA sheep showed a much smaller amount of C18:1n9t acid than heterozygous sheep, while homozygous CC animals had a significantly higher proportion. The ANXA9 gene polymorphism did not affect sheep milk’s unsaturated fatty acid ratio.

The biological function of β-LG is currently not fully understood. But Ozmen and Kul [18] demonstrated a hypothesis that this protein acts as a provider of amino acids to offspring and assists in transporting fatty acids and retinol. The results of this study showed an association between the differences in the β-LG gene and the amounts of MUFA% and PUFA% in Awassi sheep. In particular, it was found that individuals with the AA genotype of the β-LG gene had the highest levels of MUFA, while those with the AB genotype had the greatest effect on PUFA%. Mele et al. [25] did a study that showed that individuals with the β-LG-AB genotype had the lowest amounts of MCFA and the highest levels of MUFA and LCFA in their milk. There were no major changes in the PUFA of Massese lambs [26]. On the other hand, they found that the B allele was linked to higher amounts of MUFA, oleic acid, PUFA n-3, and LCFA in ewe milk from the Polish Heath breed. The findings of Tumino et al. [27] indicated that the sheep of the Valle del Belìce region do not align with prior data. In particular, the β-LG gene was found to have only a small effect on a small number of FA.

Notably, the amount of oleic acid 18:1 cis-9 (MUFA) was higher in AB milk, while the amount of linoleic acid 18:2 (n-6), alpha-linolenic acid, total trans-FA, and total PUFA was higher in BB milk. Berry et al. [28] also found that the β-LG gene had a significant impact on the amount of fat in the milk of Holstein-Friesian × Jersey crossbred cows, with BB animals having a higher percentage of fat in their milk than AA animals (*p *< 0.05).

The amount of VLCFA% was affected significantly by the AA genotype of the PRL gene. Also, it was observed that the interactions between PRL and β-LG (AAAA and BBAA) had a major impact on the amounts of MUFA and PUFA, respectively. According to Zidi et al. [29], PRL causes the production of lactose and makes it easier for the mammary gland to absorb fatty acids, lipogenesis, and triacylglycerol synthesis. Zidi et al. [29] found that a SNP called g.8948 A > T had a substantial effect on the total amount of SFA, especially LCFA-like arachidic acid (C20:0). This genomic region had an effect on MUFA as well. In homozygous, the T allele has a preference for saturation, while the A allele has a preference for unsaturation.

In a study conducted by Pegolo et al. [30], there were significant associations observed between two SNPs of the PRL gene, rs110684599 and rs211032652, and the amounts of seven specific fatty acids. The A allele of rs110684599 was linked to Rumenic acid (Va = 16.2%), C14:0 iso (Va = 3.8%), and C15:0 iso (Va = 8.4%) (Va is a measure of how much genetic variation each SNP explains (in percent)). On the other hand, the T allele of rs211032652 showed a positive relationship with C18:2 trans-9, trans-12 (Va = 1.5%), long-chain SFA [C18:0 (Va = 2.8%), and C20:0 (Va = 1.4%)], and C17:0 anteiso (Va = 6.7%).

In a study done by Zidi et al. [31], some significant correlations were found between non-synonymous allelic variation in the prolactin receptor (PRLR) and features linked to milk fatty acid contents. The amount of palmitoleic acid in milk and the genotypes of c.1201G<A and c.1355CT in the PRLR gene were found to have a statistically significant relationship. Moreover, their study found strong links between PRLR genotype and the amounts of palmitic (C16:0), elaidic (C18:1n9t), linoleic (C18:2n6c), SFA, MUFA, PUFA, and omega-3 fatty acid. It is worth mentioning that milk fatty acids and the c.1355C < T PRLR gene are related in a way that should be interpreted with care. This is due to the uneven genotype frequencies, specifically the small sample size for the CT genotypic class. The observed associations could potentially be attributed to a causal relationship between the identified missense substitutions in the cytoplasmic domain of goat PRLR. However, it is important to note that this causal effect has yet to be demonstrated at the functional level. Alternatively, these associations may have arisen due to the presence of linkage disequilibrium between the analyzed polymorphisms and one or more unidentified causal mutations.

Marchitelli et al. [31] conducted a study in which they identified additional candidate genes that have an impact on the contents of fatty acids. The study confirmed the influence of the SCD gene on the C14 desaturation index. The DGAT1 gene was found to be polymorphic only in the Jersey breed, and its impact was confirmed just on milk fat content. The study also identified three potential candidate genes. First, the FABP4 gene was found to have an influence on MCFA and LCFA in all breeds, but not on desaturation indices. Second, the FASN gene was found to affect the quantity of PUFA in the Piedmontese and Valdostana breeds. Finally, the LPL gene was found to influence fat content in the Piedmontese breed. Yakan et al. [32] reported a noteworthy inverse relationship between somatic cell count and genes associated with fatty acid production.

The specialized aspect of this study pertained to the impact of combined genotypes on the fatty acids group. The combination of genotypes serves as an indicator of the interplay between the impacts of several genes on a specific quantitative characteristic. To the best of our knowledge, there has been no prior investigation conducted on the interaction between many genes and its impact on the level of fatty acids. Our study only found a significant impact of the interaction of PRL* β-LG and the interaction of PRL*ANXA9-3 genotypes on PUFA% (Table 5). In addition, PRL* β-LG genotypes showed a significant effect on MUFA%. The study conducted by Jawasreh et al. [5] is the only one that documented the AA × BB genotype (β-LG*PRL) as having the highest percentage of fat.

## Conclusion

The important result or discovery of this study is the previously unidentified mutation C > A of the ACAA2 gene at position 7596 bp (on the size of the gene sequence). The interesting piece about this study was the effect of the combined gene genotypes on the fatty acids groups. We showed the interaction effect of PRL* β-LG genotypes on MUFA% and PUFA% as well as PRL*ANXA9-3 genotypes on PUFA%. Our study demonstrates that the presence of certain genotypes, specifically the AA and AB genotypes of β-LG, the AA genotype of ACAA2, and the CC and GG genotypes of ANXA9-3 and ANXA9-5, respectively, in sheep individuals significantly enhances the levels of fatty acids, particularly MUFA and PUFA, in their milk. Therefore, individuals carrying these specific genotypes, in addition to considering the interaction between them, should be incorporated into selection programs.

## List of abbreviations

ACAA2, acetyl-CoA acyltransferase 2; ANXA9, annexin a9; Bp, base pair; CLA, conjugated linoleic acid; LCFA, long-chain fatty acid; LDL, low-density lipoprotein; MCFA, medium-chain fatty acid; MUFA, monounsaturated fatty acid; PCR, polymerase chain reaction; PRL, prolactin; PUFA, polyunsaturated fatty acid; QTL, quantitative trait loci; RA, rumenic acid; SFA, saturated fatty acid; SNPs, single nucleotide polymorphisms; TAG, triacylglycerol; VA, vaccenic acid; VLCFA, very long chain fatty acid; β-LG, beta-lactoglobulin; RFLP, restriction fragment length polymorphism; TDM, test-day milk; SNF, solid non-fat; PCR-RFLP, polymerase chain reaction-restricted fragment length polymorphism.
